# Efficacy and Safety of Mirabegron 25 mg Versus Oral Desmopressin 120 mcg in Treatment of Primary Nocturnal Enuresis

**DOI:** 10.5152/tud.2026.26009

**Published:** 2026-05-14

**Authors:** Hosam Abdel-Fattah Abo-Elnasr, Mohamed Abdelrahman Alhefnawy, Mahmoud Farag, Mohamed Aboulfotouh El Gharably

**Affiliations:** 1 Department of Urology, Benha University, Benha, Qalubia, Egypt; 2 Department of Urology, Cairo University Kasralainy School of Medicine, Cairo, Egypt; 3 Department of Urology, Southend University Hospital, Mid and South Essex NHS Foundation Trust, Essex, UK

**Keywords:** Desmopressin, mirabegron, nocturnal enuresis

## Abstract

**Objective:**

To evaluate the effectiveness and safety of mirabegron in the management of primary nocturnal enuresis.

**Methods:**

This prospective, randomized controlled study included 150 patients randomized into 2 equal groups: desmopressin and mirabegron. All patients underwent evaluation over a 6-month period, with assessments conducted at the end of each month. Evaluations included the frequency of wet nights per month, clinical outcomes, the percentage reduction in wet nights during treatment, and relapse following treatment. The side effects of the drugs in the studied groups were also recorded.

**Results:**

At 2, 3, 4, 5, and 6 months, the mirabegron group showed a significant increase in median percentage reduction in wet nights from baseline compared with the desmopressin group. Furthermore, there was a significant improvement in clinical outcomes for the mirabegron group compared to the desmopressin group (*P* < .01*). At the 1-month follow-up after treatment, 10.7% of patients in the mirabegron group had a relapse, whereas the relapse rate in the desmopressin group was 22.7%, indicating a statistically significant difference (*P* = .049*); the relative risk of relapse with mirabegron was 0.47 (95% CI: 0.22-1.02). The mirabegron group exhibited a significant increase in maximum bladder capacity (MBC) (270.8 ± 73.80) compared to the desmopressin group (230.933 ± 67.35; *P* < .001). Both groups exhibited a statistically significant increase in MBC posttreatment compared with pretreatment (*P* < .001*). No significant difference was observed between the 2 groups regarding any of the reported side effects.

**Conclusion:**

Mirabegron is an effective treatment for nocturnal enuresis, with better outcomes, fewer relapses, and tolerable side effects.

**Main Points**

- In this prospective, randomized controlled study, 150 patients with primary nocturnal enuresis (PNE) were randomized to treatment with either mirabegron or desmopressin. Patients were monitored for side effects and other parameters over a 6-month period. Evaluations included wet-night reduction, clinical outcomes, relapse rate, and maximum bladder capacity, all assessed monthly.
- From the second to the sixth month, mirabegron demonstrated a significant reduction in wet nights and enhanced clinical outcomes relative to desmopressin (*P* < .001). Patients treated with mirabegron demonstrated more sustained control of PNE.
- Maximum bladder capacity increased significantly in both groups, with a superior improvement in the mirabegron group (270.8 ± 73.8 vs. 230.9 ± 67.3 mL, *P* < .001), and relapse 1 month after treatment was significantly lower in the mirabegron group 1 month after treatment (10.7% vs. 22.7%, *P* = .049).
- Both mirabegron and desmopressin were well tolerated, and no significant differences were observed in the reported side effects. Therefore, mirabegron may represent a safe and more effective alternative for the treatment of PNE.

## Introduction

Nocturnal enuresis (NE) is one of the most common forms of incontinence in children and frequently shows familial aggregation.^1^ It is defined as the involuntary passage of urine during sleep in a child aged 5 years or older, occurring at least twice weekly for 3 consecutive months in the absence of a congenital or acquired cause. Nocturnal enuresis is classified as primary or secondary based on whether bed dryness persists for more than 6 months.^2^ It affects approximately 15%-20% of children at the age of 5 years and is primarily attributed to delayed bladder development and function.^3^^,^^4^ Male children exhibit a higher prevalence of NE compared to female children in 50% of cases, and spontaneous resolution occurs annually in nearly 15% of affected children.^5^

Non-pharmacological management of NE includes fluid restriction and enuresis alarms. The most commonly used pharmacological agents for the treatment of NE include tricyclic antidepressants such as imipramine, the arginine vasopressin analogue desmopressin, and anticholinergic agents.^1^ However, numerous patients report that enuresis alarms are bothersome due to potential skin irritation, their propensity to disturb the sleep of other family members, and their ineffectiveness in waking the child, leading approximately 30% of patients to cease using the device.^6^ Desmopressin is approved as a first-line treatment for NE.^7^ Nevertheless, numerous studies indicate that desmopressin monotherapy is ineffective for patients with bladder storage dysfunction and is associated with a high relapse rate after treatment discontinuation. Mirabegron has been approved by the Food and Drug Administration (FDA) for the treatment of neurogenic detrusor overactivity in pediatric patients.^6^

Following the failure of desmopressin or enuresis alarms as first-line treatments, the International Children’s Continence Society has recommended the use of combination therapy for managing primary NE.^6^ Solifenacin^8^ has recently been prescribed as an alternative to oxybutynin^9^ and tolterodine,^8^ which are associated with fewer adverse effects. Oxybutynin is linked to neurological side effects, particularly cognitive impairment. A significant number of children experienced severe side effects, including headache, dry mouth, behavioral changes, flushed cheeks, constipation, and blurred vision, resulting in the early premature termination of their therapy.^10^

Mirabegron, a β3-adrenoceptor (β3-AR) agonist, is recognized as a medication that can relax the detrusor muscle and improve bladder capacity without the side effects linked to anticholinergic medications.^11^^,^^12^ Mirabegron is the inaugural β3-AR agonist utilized clinically for adult patients with overactive bladder (OAB) symptoms, demonstrating promising results.^13^ Although it is not licensed for the treatment of children with OAB, several early reviews have indicated that it is both effective and safe in this population.^14^^,^^15^

Therefore, this study aims to examine the effectiveness and safety of mirabegron for the management of primary nocturnal enuresis (PNE).

## Material and Methods

### Trial Design

This prospective, randomized, controlled, assessor-blinded study was conducted at the Urology Department of the Benha University Faculty of Medicine on patients with PNE fromNovember 2022 to November 2024. The study was approved by the local ethics and research committee of Benha university (Protocol number: RC-7-11-2022, Date: November 7, 2022), in accordance with the Declaration of Helsinki. Written informed consent was obtained from all participants prior to inclusion in the study. The authors confirm the availability and accessibility of all original data reported in this study.

The study was registered on ClinicalTrials.gov NCT05617664.

### Assessment of the Participants and Selection Criteria

Data on age and sex were collected, along with a comprehensive history that included an evaluation of daytime wetting episodes, lower urinary tract symptoms, and bowel dysfunction, specifically constipation or stool incontinence. The frequency of NE episodes per week was documented.

Monosymptomatic NE, commonly known as bedwetting, is characterized by intermittent nocturnal incontinence in the absence of daytime urinary symptoms. Non-monosymptomatic NE is characterized by the presence of NE alongside daytime lower urinary tract symptoms, recurrent urinary tract infections, and/or bowel dysfunction. Nocturnal enuresis is classified as primary when a child has not achieved consistent nighttime dryness for 6 consecutive months. Secondary NE occurs when an individual, whether a child or an adult, resumes bedwetting after a period of dryness lasting at least 6 months. This phenomenon may be linked to stressful life events, including a caregiver’s divorce, the birth of a sibling, constipation, or irregular daytime voiding patterns.^16^^,^^17^

The inclusion criteria comprised patients aged 5-15 years with primary NE, minimal daytime wetting, a frequency of wetting of at least 4 times over 4 weeks, and a normal clinical examination that ruled out neurological or urological causes of the enuresis.

Exclusion criteria comprised secondary enuresis, polysymptomatic conditions, neurologic bladder issues, neurological disorders, urinary incontinence disorders, and prior use of anti-NE drugs.

### Randomization and Allocation Concealment

Based on the assessment of patient eligibility for study participation, only patients with primary monosymptomatic NE were included. Parents were asked to complete the informed consent form. Simple random allocation was performed using sealed opaque envelopes. The envelopes were prepared by a staff member who was not involved in patient recruitment or outcome assessment. They were sequentially numbered to ensure proper allocation concealment. Each envelope included the assigned group designation and was opened solely after the participant’s enrollment in the study.

Group A: Seventy-five patients with PNE commenced on desmopressin 120 mcg oral tablets once daily at night.

Group B: Seventy-five patients with PNE commenced on mirabegron 25 mg oral tablets once daily at night.

All patients underwent evaluation for 6 months, with assessments conducted at the end of each month. The evaluations included the number of wet nights per month, clinical outcomes, the percentage reduction in wet nights during treatment, and relapse following treatment. The evaluation of maximum bladder capacity (MBC) was conducted before and after treatment. The side effects of the studied groups were recorded.

### Measurement of Bladder Capacity

Bladder capacity was assessed using 48-hour frequency/volume (F/V) charts and confirmed by uroflowmetry. Maximum voiding volume was defined as the largest volume on the F/V chart, excluding first morning voids. Uroflowmetry was performed during a single outpatient visit, starting from the second morning void and repeated twice based on the normal urge to void after voluntary fluid intake. Post-void residual (PVR) was measured by transabdominal ultrasound. Maximum bladder capacity from uroflowmetry was calculated as the sum of the largest voided volume and its corresponding PVR.^18^

### Response

A complete cure was defined as no more than 1 wet night per month. Response was defined as a reduction of 90% or more in wet nights, partial response as a reduction of 50%-89%, and no response as little to no treatment effect.

### Relapses

Relapse was defined as the recurrence of more than 1 wet night during the first month after the end of the treatment period, corresponding to the seventh month of follow-up in the current study.

### Outcomes

The primary outcome was to assess the treatment’s efficacy and relapse rate posttreatment, while the secondary outcome focused on the safety profile of each drug by examining adverse effects associated with its use.

### Sample Size Calculation

According to Esteghamati et al,^19^ the treatment group of desmopressin plus tolterodine demonstrated a 20% failure to respond to treatment, compared with 50% in the desmopressin plus indomethacin group. Therefore, the present study required a sample size of 58 patients per group, for a total of 116, assuming equal group sizes, to achieve a power of 90% at a two-sided significance level of 5%. The sample size was increased to 150 patients (75 per group) to compensate for an anticipated 30% dropout rate. The sample size was calculated using G*power software 3.1.9.2.

### Data Management and Analysis

Data entry, coding, and analysis were conducted using IBM SPSS Statistics for Windows, Version 28.0. (IBM SPSS Corp.; Armonk, NY, USA) . Data from this study included both quantitative (expressed as means ± SD, median, minimum, and maximum) and qualitative (expressed as numbers and percentages).

Tests of significance used were the following:

- A post-hoc power analysis was performed based on a sample size of 75 patients per group. The mean percentage reduction in wet nights after 6 months was 72.62% ± 27.58% in the desmopressin group compared to 88.87% ± 16.05% in the mirabegron group. This represents a Cohen’s *d* effect size of 0.72, which is categorized as a medium-to-large effect. With a two-sided alpha of 0.05, the study achieved a statistical power of 0.992 (99.2%). These findings indicate that the study was adequately powered to detect the significant clinical improvement observed between the 2 groups.
- Tests of the Kolmogorov–Smirnov and Shapiro–Wilk were used to test the normality of the distributions of quantitative variables.
- According to the normality of the distribution, the Mann–Whitney (*U*) test was used to test the significance of nonparametric numerical data between 2 groups.
- Pearson’s chi-square (*χ*^2^) was used to test the significance of the association between 2 categories.
- The Friedman (*χ*^2^_(F)_) test was used to compare 3 repeated measures of nonparametric quantitative data within the same group, and the Wilcoxon signed-rank test (*Z*) was used for pair-wise comparisons between 2 repeated measures.
- The level of statistical significance was set at 95%; therefore, *P*-values > .05 were considered non-significant, and *P*-values < .05 were considered statistically significant.

## Results

This study included 150 cases, divided into 2 groups: 75 in the desmopressin group and 75 in the mirabegron group. Participants’ ages ranged from 5 to 15 years, with a mean age of 9.17 ± 2.92 years in the desmopressin group and 9.12 ± 2.73 years in the mirabegron group. The desmopressin group comprised 58.7% males, while the mirabegron group included 56.0% males. No significant differences were observed between the 2 study groups for age or sex.

**Figure 1.**
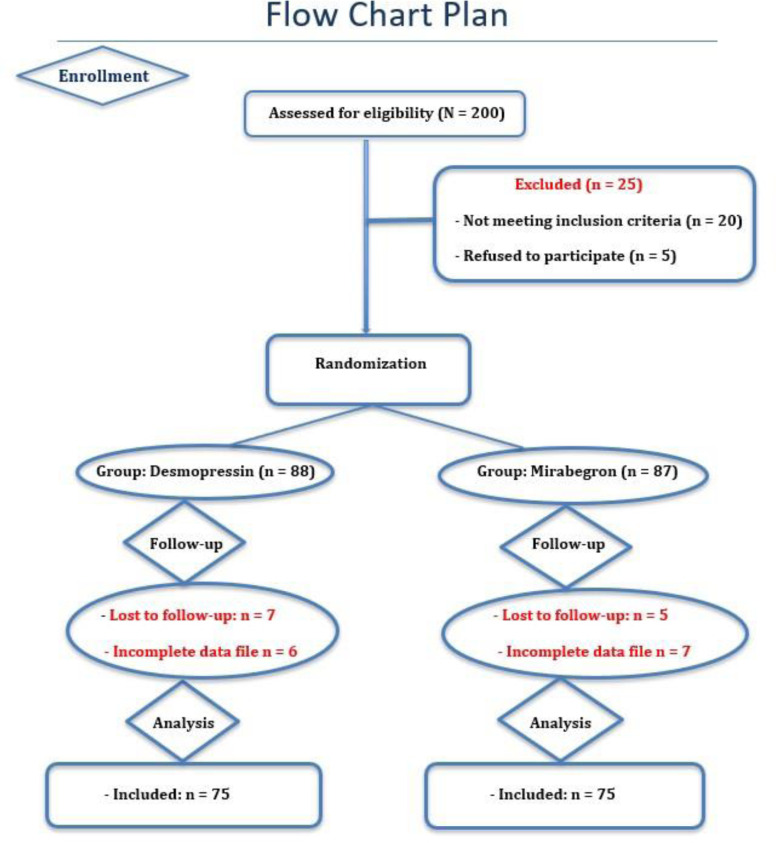
Flow chart of participants through each stage of the study.

Figure 1 depicts the flow diagram and analysis of the study outcomes according to the Consolidated Standards of Reporting Trials.^20^

Regarding baseline and monthly follow-up measurements of wet nights, there was no significant difference between the 2 groups. However, the mirabegron group showed a significantly greater reduction than the desmopressin group at all monthly follow-up assessments at 1, 2, 3, 4, 5, and 6 months (Table 1).

The percentage reduction in wet nights per month from pretreatment values, monthly follow-up clinical outcomes, and relapses after treatment in the study groups are presented in Table 2.

Regarding relapse after 1 month follow-up posttreatment, 10.7% of the mirabegron group suffered from relapse compared to 22.7% in the desmopressin group, with a slightly significant difference, *P* = .049* (Table 2). The absolute risk reduction was 12.0%, and the calculated relative risk of relapse with mirabegron was 0.47 (95% CI: 0.22-1.02), suggesting that patients receiving mirabegron had approximately a 53% lower risk of relapse compared with those receiving desmopressin.

There was no significant difference in baseline MBC between the mirabegron and desmopressin groups. Both groups showed a significant increase in MBC after treatment compared to before treatment, p < 0.001* (Table 3). There was no significant difference between the 2 groups in all reported side effects (Table 4).

## Discussion

Various therapeutic options are available for treating NE, including behavioral modification, alarm therapy, and pharmacological therapy, which can be beneficial in promoting adherence to behavioral therapy among children and in aiding early symptom management. Antimuscarinics alone have not proven effective.^21^ Mirabegron, the first β3-adrenergic receptor agonist approved for clinical use, offers a promising alternative. As a novel “first-in-class” agent, mirabegron has demonstrated efficacy in managing OAB symptoms in adults.^13^ More recently, it became the first β3-adrenergic receptor agonist approved for pediatric use, following its 2021 FDA approval for the treatment of neurogenic detrusor overactivity in children aged ≥3 years.^22^

Ghanavati et al^23^ investigated the tolerability and effectiveness of desmopressin alone, desmopressin combined with tolterodine, and desmopressin combined with solifenacin in the treatment of primary NE. At baseline, the mean frequency of enuretic episodes was 30.09/month in the desmopressin monotherapy group, 33.01/month in the desmopressin–tolterodine group, and 40.40/month in the desmopressin–solifenacin group. After 3 months, these frequencies decreased to 12.32, 4.55, and 1.50 episodes per month, respectively, demonstrating the superior efficacy of combination therapy with desmopressin and antimuscarinics.^23^ In the current study, relapse occurred in 10.7% of the mirabegron group and 22.7% of the desmopressin group, with a slight, significant difference (*P* = .049*). This is in line with earlier studies that reported that children receiving desmopressin therapy respond in 60%-70% of cases after 3 months of treatment.^24,25^ Additionally, approximately 50% of patients receiving desmopressin monotherapy achieved full responses and relapsed after treatment was discontinued, highlighting a limitation of desmopressin monotherapy in maintaining long-term remission.^24,^^25^

Van Veen et al^26^ reported that mirabegron yields long-term improvement in bladder function among pediatric patients with therapy-refractory neurogenic lower urinary tract dysfunction, with benefits maintained for over 2 years without significant adverse effects.

The results indicated a significant decrease in the number of wet nights per month in the mirabegron group relative to the desmopressin group across all monthly follow-ups from 1 to 6 months. The lasting impact of mirabegron on bladder compliance and continence, as previously reported by Van Veen et al,^26^ was demonstrated by the significant reduction in wet nights in the mirabegron group during the 6-month follow-up in this study. Following 1 month of treatment, a reduction in wet nights from baseline was noted in both the mirabegron and desmopressin groups. The difference between the 2 groups was not statistically significant, *P* = .244, suggesting a similar initial response regarding symptom improvement in the early phase of therapy. Between the second and sixth months of follow-up, the mirabegron group exhibited a significantly greater median percentage reduction in wet nights compared to the desmopressin group at all assessed time points. After 6 months, 65.3% of patients in the mirabegron group achieved complete cure, and 8% showed partial response, versus 42.7% and 5.3%, respectively, in the desmopressin group *P* < .001*), confirming the superior efficacy of mirabegron.

Ghanavati et al^23^ found that 45.46%, 59.09%, and 63.63% of patients in the desmopressin treatment group achieved complete remission at 1, 2, and 3 months of treatment, respectively. Nonetheless, the rates of complete remission rose to 75%, 80%, and 85% in the groups receiving desmopressin, tolterodine, and both, respectively. The rates of complete remission increased to 75%, 80%, and 85% in the groups treated with desmopressin, tolterodine, and both, respectively. The evaluation of the group treated with desmopressin and solifenacin indicated higher rates of complete remission: 85% after 1 month, 90% after 2 months, and 95% after 3 months.^23^

Munding et al^27^ administered tolterodine (1 mg twice daily) to a cohort of 30 children, aged 4-17 years, diagnosed with primary dysfunctional voiding. Among these, 40% attained partial remission, while 33% attained complete remission. Only 13% reported drug-related side effects, with 1 child discontinuing treatment as a result of diarrhea. None displayed the common adverse effects associated with oxybutynin, including flushing, hyperpyrexia, or photosensitivity.^27^

Austin et al^21^ compared the efficacy of desmopressin combined with tolterodine versus desmopressin alone in 41 patients with lower urinary tract dysfunction and desmopressin-resistant NE. Patients were randomized into 2 groups and treated for 4 weeks: 1 group received desmopressin plus placebo, while the other received desmopressin plus tolterodine. After 1 month, the combination therapy group demonstrated a significantly higher mean number of dry nights, with bedwetting reduced by 66% compared to desmopressin monotherapy.^21^

Zaffanello et al^28^ evaluated the efficacy of tolterodine in children with treatment-resistant enuresis in a 5-week trial involving participants who had not responded to desmopressin and alarm therapy. The children were divided into 3 groups receiving placebo, tolterodine (1-2 mg daily), or imipramine (25-50 mg at bedtime). Among 25 children, the mean number of wet nights was 7.8 ± 5.1 for placebo, 10.4 ± 3.9 for tolterodine, and 11 ± 3.9 for imipramine.^28^ Previous studies indicate that antimuscarinics, including tolterodine, demonstrate limited effectiveness when administered as monotherapy for pediatric dysfunctional voiding and treatment-resistant enuresis. While some cases have reported partial and complete remission, numerous patients do not attain a prolonged therapeutic response with antimuscarinic therapy alone. This highlights the necessity for alternative strategies or combination therapies to improve treatment outcomes.

Fryer et al^29^ were the first to describe the clinical application of mirabegron in pediatric patients with treatment-resistant OAB. In their observational study, they reported that approximately 70% of patients who received mirabegron therapy for 6 months or longer experienced significant improvements in bladder symptoms, including reductions in urgency and frequency, with a favorable safety profile. These findings highlighted mirabegron as a promising alternative for children unresponsive to conventional antimuscarinic treatments.^29^

In this study, there was no significant difference in baseline MBC between the mirabegron and desmopressin groups. Following treatment, both groups showed significant within-group increases in MBC (*P* < .001*), based on mean values calculated across all patients in each group, reflecting a generalized treatment effect. However, the magnitude and clinical relevance of improvement varied significantly between groups. The mirabegron group exhibited a significant increase in the proportion of patients with normal bladder capacity, rising from 45.3%-82.7%. Conversely, the proportion of patients with reduced capacity decreased from 54.7%-17.3% (*P* < .001*), demonstrating both statistical and clinical efficacy. The desmopressin group exhibited a modest, non-significant enhancement in capacity distribution, with minimal change in the reduced-capacity subgroup. The findings indicate that mirabegron is more effective in restoring bladder storage function in patients with NE.

This aligns with the findings of a retrospective study conducted by Kim et al,^30^ which compared the efficacy and tolerability of mirabegron and solifenacin in children with idiopathic OAB. The primary analysis included 45 patients: 29 in the solifenacin group and 16 in the mirabegron group. Both treatment groups showed significant improvements in the age-adjusted bladder capacity ratio. The solifenacin group exhibited an increase from 0.71 to 0.96 (*P* < .001), whereas the mirabegron group improved from 0.57 to 0.97 (*P* = .002) after treatment. The study found that lower baseline bladder capacity was significantly correlated with a positive response to medication (odds ratio 7.41; 95% CI not reported; *P* = .044), indicating that patients with greater impairment in bladder capacity prior to treatment were more likely to experience benefits.^30^ This finding is significant to the current study’s results, as a substantial proportion of patients in the current study population had reduced baseline bladder capacity. This illustrates the diverse etiology of NE, highlighting the significant role of functional bladder capacity. This subgroup represents a substantial segment of the NE population and is a critical focus for therapies designed to enhance bladder capacity. Following treatment with mirabegron, these patients exhibited significant improvement, as evidenced by a marked increase in normal bladder capacity. The observations support the clinical relevance of baseline bladder capacity as a predictor of treatment response in pediatric lower urinary tract symptoms.

Consistent with the present study, Nasution et al^31^ further support the safety and tolerability of mirabegron, a β3-adrenoceptor agonist that facilitates detrusor muscle relaxation during the bladder storage phase, in pediatric patients with neurogenic bladder dysfunction and NE. The present study highlights the favorable side-effect profile associated with mirabegron therapy. No anticholinergic-type adverse effects, such as dry mouth, constipation, or cognitive disturbances, were noted in the mirabegron group, which may hold significance for pediatric populations. The absence of these common anticholinergic side effects enhances tolerability and promotes long-term adherence to treatment.

This study has limitations, notably its single-center design, which may restrict the generalizability of the findings. The study offers important insights into the efficacy of mirabegron for primary NE; however, results may be affected by center-specific factors, including patient selection criteria, local clinical practices, and referral patterns. These factors may influence treatment outcomes and response rates. Larger multi-center studies involving diverse patient populations and clinical settings are necessary to validate these findings across a wider range of patients. This study is limited by a relatively short follow-up period. The results indicate efficacy during the treatment phase; however, an extended posttreatment follow-up of up to 6 months would yield more definitive evidence regarding the durability of response and the potential for relapse. Future research should incorporate extended posttreatment follow-up to better assess the long-term efficacy and safety of mirabegron in pediatric patients with primary NE. A head-to-head comparison is advantageous; however, including a placebo group or behavioral therapy alone may provide context for the overall benefits of pharmacological treatment.

It is advisable to investigate further therapeutic alternatives, including the comparison of mirabegron with combination regimens involving anticholinergics, to determine the most effective and safest treatment strategies for PNE.

Mirabegron may serve as a safe and effective monotherapy for children with primary NE. This study indicates a positive effect and a reduction in relapse rates. This mechanism improves bladder storage and maintains antidiuretic pathways, potentially offering clinical advantages for patients with reduced bladder capacity.

## Figures and Tables

**Table 1.** Baseline and Follow-Up of Wet Nights Per Month in the Study Group

**Variable**	**Desmopressin Group (n = 75)**	**Mirabegron Group (n = 75)**	**Test of Significance, *U***	***P***
Baseline wet nights per month Mean ± SD Median (min-max)	18.837 ± 4.46 19 (12-27)	17.44 ± 5.97 17 (7-30)	2408.0	.127
One month after treatment Mean ± SD Median (min-max)	7.24 ± 5.45 6 (1-20)	4.63 ± 2.873 4 (2-14)	2266.00	.038*
Two months after treatment Mean ± SD Median (min-max)	5.41 ± 5.104 3 (0-17)	2.07 ± 3. 310 1 (0-14)	1602.0	<.001*
Three months after treatment Mean ± SD Median (min-max)	4.6 ± 5.17 1 (0-16)	1.53 ± 3.22 0 (0-13)	1874.5	<.001*
Four months after treatment Mean ± SD Median (min-max)	5.107 ± 5.40 1 (0-17)	2.13 ± 3.50 1 (0-14)	1966.0	<.001*
Five months after treatment Mean ± SD Median (min-max)	5.52 ± 5.73 2 (0-18)	1.947 ± 3.16 1 (0-14)	1731.0	<.001*
Six months after treatment Mean ± SD Median (min-max)	5.48 ± 5.99 2 (0-19)	1.987 ± 3.23 1 (0-14)	*U* = 1982.0	<.001*
Test of Significance	*χ*^2^_(F) _= 287.478	*χ*^2^_(F) _= 335.008		
*P*	<.001*	<.001*		
Pair-wise comparison compared to baseline	P1, P2, P3, P4, P5, P6 <.001*	P1, P2, P3, P4, P5, P6 <.001*		
Pair-wise comparison compared to after 3 months	P1, P2, P4, P5, P6 <.001*	P1, P2, P4, P5, P6 <.001*		
Pair-wise comparison compared to after 6 months	P1, P3, P4 <.01* P2, P5 >.05	P1, P3<.01* P2, P4, P5 >.05		

Pair-wise comparison *P*-values are expressed as *Z* using the Wilcoxon signed-rank test. *Significant at *P* < .05; non-significant at *P* > .05.

P1: comparison versus after 1 month; P2: comparison versus after 2 months; P3: comparison versus after 3 months; P4: comparison versus after 4 months; P5: comparison versus after 5 months; P6: comparison versus after 6 months.

Max, maximum; Min, minimum; *U*, Mann–Whitney test; *χ*^2^_(F)_, Friedman test.

**Table 2.** Monthly Follow-Up of Clinical Outcomes, Percentage Reduction in Wet Nights During Treatment, and Relapse After Treatment in the Study Groups

**Month**	**Variable**	**Desmopressin (n = 75)**	**Mirabegron (n = 75)**	**Test**	***P***
After 1 month	Reduction %	Mean ± SD: 63.14 ± 24.21 Median: 66.67 (20-94.74)	Mean ± SD: 73.57 ± 12.91 Median: 76.47 (33.3-86.96)	*U* = 2503.0	.244
	Clinical outcome	Complete cure: 11 (14.7%) Response: 2 (2.7%), Partial response: 35 (46.6%) No response: 27 (36.0%)	Complete cure: 0 (0%) Response: 0 (0%) Partial response: 69 (92%) No response: 6 (8%)	FEX = 39.203	<.001*
After 2 months	Reduction %	Mean ± SD: 72.49 ± 23.78 Median: 84.21 (32-100)	Mean ± SD: 89.72 ± 16.01 Median: 94.12 (33.33-100)	*U* = 1539.5	<.001*
	Clinical outcome	Complete cure: 24 (32%) Response: 4 (5.3%) Partial response: 28 (37.3%) No response: 19 (25.3%)	Complete cure: 52 (69.3%) Response: 6 (8%) Partial response: 11 (14.7%) No response: 6 (8%)	*χ*^2^ = 24.886	<.001*
After 3 months	Reduction %	Mean ± SD: 76.97 ± 24.19 Median: 91.67 (36-100)	Mean ± SD: 92.47 ± 15.71 Median: 100 (38-100)	*U* = 1869.0	<.001*
	Clinical outcome	Complete cure: 38 (50.6%) Response: 2 (2.7%) Partial response: 18 (24%) No response: 17 (22.7%)	Complete cure: 61 (81.4%) Response: 4 (5.3%) Partial response: 4 (5.3%) No response: 6 (8%)	FEX = 20.411	<.001*
After 4 months	Reduction %	Mean ± SD: 74.31 ± 25.12 Median: 91.67 (31.25-100)	Mean ± SD: 88.93 ± 16.95 Median: 93.75 (33.33-100)	*U* = 2095.0	.007*
	Clinical outcome	Complete cure: 38 (50.7%) Response: 2 (2.7%), Partial response: 16 (21.3%) No response: 19 (25.3%)	Complete cure: 49 (65.3%) Response: 8 (10.7%) Partial response: 12 (16%) No response: 6 (8%)	*χ*^2^ = 12.322	.004*
After 5 months	Reduction %	Mean ± SD: 72.16 ± 26.36 Median: 88.89 (25-100)	Mean ± SD: 89.60 ± 15.46 Median: 94.12 (33.3-100)	*U* = 1708.0	<.001*
	Clinical outcome	Complete cure: 28 (37.3%) Response: 4 (5.3%) Partial response: 24 (32%) No response: 19 (25.4%)	Complete cure: 53 (70.7%) Response: 4 (5.3%) Partial response: 14 (18.7%) No response: 4 (5.3%)	FEX = 20.535	<.001*
After 6 months	Reduction %	Mean ± SD: 72.62 ± 27.58 Median: 89.47 (24-100)	Mean ± SD: 88.87 ± 16.05 Median: 92.86 (33.3-100)	*U* = 2089.0	.006*
	Clinical outcome	Complete cure: 32 (42.7%) Response: 4 (5.3%) Partial response: 14 (18.7%) No response: 25 (33.3%)	Complete cure: 49 (65.3%) Response: 6 (8%) Partial response: 14 (18.7%) No response: 6 (8%)	*χ*^2^ = 15.613	<.001*
Relapse	N (%)	17 (22.7)	8 (10.7)	*χ*^2^ = 3.888	.049*

FEX, Fisher’s exact chi-square test; Max, maximum; Min, minimum; *U*, Mann–Whitney test; *χ*^2^, chi-square test.

*Significant at *P* < .05.

**Table 3.** Maximum Bladder Capacity Before and After Treatment in the Study Groups

**Variable**	**Desmopressin Group (n = 75)**		**Mirabegron Group (n = 75)**		**Test of Significance**	***P***
MBC before					*U* = 2753.0	.823
Mean ± SD	227.67 ± 68.91		227.33 ± 76.49			
Median (min-max)	220 (115-370)		210 (115-400)			
MBC after					*U* = 1949.5	<.001*
Mean ± SD	230.933 ± 67.35		270.8 ± 73.80			
Median (min-max)	227 (115-370)		260 (145-430)			
Test of significance	*Z* = 4.474		*Z* = 7.556			
*P*	.000**		.000**			
MBC before	Normal	Reduced	Normal	Reduced	*χ*^2^ = 0.108	.742
N (%)	32 (42.7)	43 (57.3)	34 (45.3)	41 (54.7)	*U* = 12.005	.007*
Mean ± SD	330.71 ± 63.06	173.12 ± 62.3	331.52 ± 78.8	178.23 ± 71.2		
Median (min-max)	327 (280-370)	170 (115-290)	330 (250-400)	175 (115-280)		
MBC after					*χ*^2^ = 5.075	.024*
N (%)	38 (50.7)	37 (49.3)	62 (82.7)	13 (17.3)	*U* = 17.713	<.001*
Mean ± SD	335.71 ± 61.2	176.32 ± 67.4	362.61 ± 75.1	215.21 ± 37.2		
Median (min-max)	335 (280-370)	175 (115-300)	365 (250-430)	210 (145-290)		
*P* (McNemar test)	.063		<.001*			
*P* (Wilcoxon signed-rank test)	Normal *P* = .046*		Normal *P* = .002*			
	Reduced *P* = .049*		Reduced *P* < .001*			

MBC, maximum bladder capacity; *U*, Mann–Whitney test; *χ*2, chi-square test; * significant

**Table 4.** Side Effects Reported in the Studied Groups

**Variable**	**Desmopressin Group (n = 75)**		**Mirabegron Group (n = 75)**		**Test of Significance**	***P***
	**N**	**%**	**N**	**%**		
Side effects	11	14.7	16	21.3	*χ*^2^ = 1.129	.396
Abdominal pain	0	0	2	2.7	FEX = 2.027	.497
Blurred vision	0	0	4	5.3	FEX = 4.110	.120
Constipation	3	4	0	0	FEX = 3.061	.245
Difficulty to fall asleep	2	2.7	0	0	FEX = 2.027	.497
Dry mouth	2	2.7	0	0	FEX = 2.027	.497
Fatigue	2	2.7	2	2.7		
Headache	0	0	2	2.7	FEX = 2.027	.497
Nasopharyngitis	0	0	2	2.7	FEX = 2.027	.497
Nausea	2	2.7	4	5.3	FEX = 0.694	.681

Data are expressed as mean ± SD, median (Min-Max).

FEX, Fisher’s exact chi-square test.

*Significant at *P* < .05.

## Data Availability

The data that support the findings of this study are available on request from the corresponding author.
